# Angiotensin II type 1 receptor antagonists alleviate muscle pathology in the mouse model for laminin-α2-deficient congenital muscular dystrophy (MDC1A)

**DOI:** 10.1186/2044-5040-2-18

**Published:** 2012-09-03

**Authors:** Sarina Meinen, Shuo Lin, Markus A Ruegg

**Affiliations:** 1 Biozentrum, University of Basel, Basel, Switzerland

**Keywords:** TGF-β, Smad2/3, Notexin, Skeletal muscle, Fibrosis, Muscle regeneration, losartan, Angiotensin II

## Abstract

**Background:**

Laminin-α2-deficient congenital muscular dystrophy (MDC1A) is a severe muscle-wasting disease for which no curative treatment is available. Antagonists of the angiotensin II receptor type 1 (AT1), including the anti-hypertensive drug losartan, have been shown to block also the profibrotic action of transforming growth factor (TGF)-β and thereby ameliorate disease progression in mouse models of Marfan syndrome. Because fibrosis and failure of muscle regeneration are the main reasons for the severe disease course of MDC1A, we tested whether L-158809, an analog derivative of losartan, could ameliorate the dystrophy in *dy*^*W*^*/dy*^*W*^ mice, the best-characterized model of MDC1A.

**Methods:**

L-158809 was given in food to *dy*^*W*^*/dy*^*W*^ mice at the age of 3 weeks, and the mice were analyzed at the age of 6 to 7 weeks. We examined the effect of L-158809 on muscle histology and on muscle regeneration after injury as well as the locomotor activity and muscle strength of the mice.

**Results:**

We found that TGF-β signaling in the muscles of the *dy*^*W*^*/dy*^*W*^ mice was strongly increased, and that L-158809 treatment suppressed this signaling. Consequently, L-158809 reduced fibrosis and inflammation in skeletal muscle of *dy*^*W*^*/dy*^*W*^ mice, and largely restored muscle regeneration after toxin-induced injury. Mice showed improvement in their locomotor activity and grip strength, and their body weight was significantly increased.

**Conclusion:**

These data provide evidence that AT1 antagonists ameliorate several hallmarks of MDC1A in *dy*^*W*^*/dy*^*W*^ mice, the best-characterized mouse model for this disease. Because AT1 antagonists are well tolerated in humans and widely used in clinical practice, these results suggest that losartan may offer a potential future treatment of patients with MDC1A.

## Background

Losartan (Cozaar^®^; Merck Sharpe & Dohme, Whitehouse Station, NJ, USA), is widely used in clinics to treat hypertension, cardiomyopathy and chronic renal disease. It is very well tolerated by all age groups. Losartan is a potent inhibitor of angiotensin II receptor type 1 (AT1) and thus lowers the blood pressure by directly causing vasodilation, and by reducing secretion of vasopressin and aldosterone. Activation of AT1 by angiotensin II results in the production of thrombospondin (TSP)-1, which has been shown to be a key regulator of TGF-β activation [1,2]. Therefore, AT1 inhibition and subsequent reduction of TSP-1 production has been shown to block TGF-β activation [1].

TGF-β is a cytokine whose activity is known to inhibit myoblast differentiation, promote fibrosis [3], and impair regeneration capacity in muscle [4]. TGF-β signals via phosphorylation of Smad2/Smad3, which then form a complex with Smad4 that translocates into the nucleus, where it modulates transcription [5]. TGF-β also activates the mitogen-activated protein kinases (MAPKs), including extracellular signal-regulated kinase (ERK)1/2, Jun kinase (JNK) and p38, which then can regulate Smad proteins or transcription factors [6,7]. Raised TGF-β levels and activity were shown to contribute strongly to the phenotype of several diseases [8-10]. For example, Marfan syndrome (MFS), which is caused by mutations in the gene encoding fibrillin-1 [11], is characterized by increased fibrosis and impaired muscle regeneration [1]. Beside structural functions, fibrillin-1 negatively regulates TGF-β signaling [9,10]. Consequently, mutations in fibrillin-1 lead to increased TGF-β levels in muscles of patients with MFS and in mouse models of MFS (*Fbn1*C1039G/+) [1,12]. In addition, increased TGF-β levels were found in muscles of Duchenne muscular dystrophy patients and *mdx* mice [8,10], and in old mice suffering from sarcopenia [13]. Importantly, when *Fbn1*C1039G/+ and *mdx* mice were treated with losartan, AT1-mediated TGF-β signaling was inhibited, decreased fibrosis, normalized muscle architecture, and improved muscle function and regeneration [1,14,15]. In mice with sarcopenia, losartan improved muscle remodeling after injury, and protected muscle from disuse-induced atrophy [13].

Laminin-α2-deficient congenital muscular dystrophy (MDC1A) is a severe muscle-wasting disease that leads to death in early childhood [16]. MDC1A is caused by mutations in the gene encoding the laminin-α2 chain, which is needed to form the heterotrimeric laminin-211, the main laminin isoform in the basement membranes of muscle and peripheral nerve [17]. In MDC1A, absence of laminin-211 disrupts the linkage of the basement membrane to the underlying cell layer, and interrupts intracellular signaling. Consequently, muscle fibers degenerate upon contraction as a result of the poor mechanical stability, fail to regenerate properly [18,19], and often undergo apoptosis [18,20]. The muscles of patients with MDC1A and of mouse models of MDC1A are characterized by extensive fibrosis, marked variation in muscle fiber size, and a greatly impaired ability of muscle to regenerate [19-25].

Over the last 10 years, various studies have been carried out on MDC1A mouse models to test potential treatment options. To date, transgenic expression of laminin-α1, a homolog of laminin-α2, in laminin-α2-deficient *dy*^*3K*^*/dy*^*3K*^ mice has shown the highest efficacy in restoring muscle function [26,27]. Similarly, a very profound restoration of muscle is achieved by transgenic expression of mini-agrin, a miniaturized form of the basement membrane component agrin in *dy*^*3K*^*/dy*^*3K*^[21] and *dy*^*W*^*/dy*^*W*^ mice [19,25]. Interestingly, expression of mini-agrin by systemic delivery of recombinant adeno-associated virus (AAV) has also been shown to have a strong ameliorating effect in *dy*^*W*^*/dy*^*W*^ mice [28].

Although these genetic therapies are interesting, the translation of such approaches into clinical practice remains difficult. Hence, several pharmacological approaches have been tested, which would eventually allow clinical treatment options. These include inhibition of apoptosis in *dy*^*W*^*/dy*^*W*^ mice [29-32] and interference with proteasomal and autophagy-mediated degradation of proteins [33,34], Halofuginone, an analog of a plant alkaloid that blocks TGF-β-mediated collagen synthesis, was tested in *dy*^*2J*^*/dy*^*2J*^ mice, which represent a much milder form of MDC1A that is caused by the partial loss of laminin-211 [35]. In these mice, halofuginone was shown to inhibit Smad3 phosphorylation downstream of TGF-β activation and to prevent progression of fibrosis, resulting in an amelioration of the dystrophic phenotype [36]. Likewise, in *dy*^*2J*^/*dy*^*2J*^ mice, losartan was shown to inhibit TGF-β signaling, improve grip strength, and reduce fibrosis [37]. Besides the mouse data, there is evidence that TGF-β levels are increased in muscles of patients with MDC1A [38].

Therefore, we aimed to test the effect of the AT1 inhibitor L-158809, a potent derivative of losartan, in the severe *dy*^*W*^*/dy*^*W*^ mouse model for MDC1A. We found that AT1-mediated TGF-β signaling contributes to the pathology in MDC1A, and that L-158809 treatment reduces TGF-β levels. Fibrosis was reduced and several histological hallmarks of disease were improved. Importantly, L-158809 supported successful regeneration in *dy*^*W*^*/dy*^*W*^ muscles, and improved body weight, grip strength, and locomotor activity. Taking into consideration the fact that losartan is already in clinical use and is well tolerated in all age groups, this treatment could proceed to clinical testing quickly and, might be a supportive treatment for patients with MDC1A in the near future.

## Methods

### Ethics approval

All procedures were approved by the veterinary commission of the Canton Basel-Stadt, and were performed in accordance with the Swiss regulations for animal experimentation.

### Treatment of *dy*^*W*^*/dy*^*W*^ mice with the angiotensin II type 1 receptor antagonist L-158809

*Dy*^*W*^/*dy*^*W*^ mice served as the mouse model for MDC1A, and were genotyped as previously described [24]. Age-matched wild-type (WT) mice served as the control group. To ensure optimal access of *dy*^*W*^/*dy*^*W*^ mice to water and food, cages were supplied with long-necked water bottles, and wet food was placed inside the cage.

Mice were treated with L-158809 (5,7-dimethyI-2-ethyI-3-[[2"-(−1 H-tetrazol-5yI)[1,1]-bi-phenyl-4-yl]-methyl]-3 H-imidazo[4,5-b]pyridine: generously provided by Merck Sharp & Dohme Research Laboratories, West Point, PA, USA). L-158809 is a potent orally bioavailable angiotensin II type 1 receptor blocker, and constitutes a more potent, chemically modified derivative of losartan (DuP-553; Merck) [39]. L-158809 was solubilized in 15% NaHCO_3_, then 0.6 g/L of L-158809 and 4% sucrose was added to the drinking water. This solution was given as drinking water and was used for the preparation of the wet food. L-158809 treatment started at the age of 3 weeks and was continued until the animals were killed by CO2 asphyxiation at the age of 6 or 7 weeks. L-158809 treatment did not influence the body weight, muscle function, or muscle architecture of WT mice (data not shown).

### Masson trichrome and immunostaining

The triceps brachii and diaphragm muscles were immersed in 7% gum Tragacanth (Sigma-Aldrich, St. Louis, MO, USA) and rapidly frozen in liquid nitrogen-cooled isopentane at −150°C). Cross-sections, 12 μm in thickness, were cut using a cryostat (Leica CM 1950; Leica Biosystems, Nussloch, Germany) and collected on slides (SuperFrost^®^ Plus; Thermo Fisher Scientific Inc., Rockford, IL, US). Masson"s trichrome staining [40] was performed to visualize collagenous tissue, using a commercial kit (HT-15; Trichrome Stain Kit; Sigma-Aldrich). The antibodies used for stainings were purchased from commercial sources as follows. Monoclonal rabbit anti-mouse TGF-β (56E4, CST #3709), monoclonal rabbit anti-human phospho-Smad2(Ser465/467)/Smad3(Ser423/425) antibody (CST #9510) (both Cell Signaling Technology, Beverly, MA, USA) polyclonal rabbit anti-human TSP 1 (LS-C26356; Lifespan Biosciences, Inc., Seattle, WA, USA), polyclonal rabbit anti-human periostin (RD181045050; BioVendor LLC, Candler, NC, USA), monoclonal rat anti-mouse F4/80 (ab6640; Abcam, Cambridge, MA, USA), monoclonal mouse anti-rat developmental myosin heavy chain (dMyHC) (NCL-MHCd; Novocastra, Norwell, MA, USA), monoclonal rat anti-mouse laminin-γ1 chain (MAB1914; Chemicon (now EMD Millipore, Billerica, MA, USA)). Membrane-bound and extracellular epitopes were visualized with Alexa-488-conjugated wheatgerm agglutinin (WGA) (Molecular Probes, Eugene, OR, USA). Depending on the source of the primary antibody, the appropriate Cy3-conjugated (Jackson ImmunoResearch Laboratories, West Grove, PA, USA) or Alexa Fluor^®^ 488-conjugated (Molecular Probes) secondary antibodies or tetramethyl rhodamine isothiocyanate (TRITC)-labeled streptavidin were used for visualization. DAPI (4",6"-diamidino-2-phenylindole hydrochloride) was used to stain nuclei. Pictures of stained cross-sections were taken using a fluorescence microscope (DM5000B; Leica, Heerbrugg, Switzerland), a digital camera (F-View), and analySIS^®^ software (both Soft Imaging Systems Corp, Lakewood, CO, USA).

### Histological quantifications

The triceps brachii and diaphragm muscles were chosen for histological analysis to exclude muscles that are affected by the secondary atrophic effect resulting from hind-limb paralysis, which is caused by demyelination of the peripheral nerve [41]. Mid-belly cross-sections were analyzed. Nuclear accumulation of pSmad2/3 was counted in four corresponding squares on cross-sections stained with phospho-Smad2 (Ser465/467)/Smad3 (Ser423/425) and DAPI. Fibrosis was quantified by measuring the collagenous area on entire cross-sections stained with Masson trichrome, and was normalized to muscle area. F4/80 staining allowed counting of macrophages in four corresponding squares in the triceps and in the entire cross-section of the diaphragm. The muscle fiber-size distribution was quantified on entire WGA-stained cross-sections using the minimum distance of parallel tangents at opposing particle borders (minimal Feret’s diameter) as described previously [42]. The variance coefficient of the muscle fiber size was defined as follows: variance coefficient = (standard deviation of the muscle fiber size ÷ mean muscle fiber size) × 1000. Normalization of the number of fibers in each Feret class of 5 μm was based on the total fiber number per muscle. Fibers with centrally located nuclei (centrally nucleated fibers; CNF) were counted in the entire WGA/DAPI-stained cross-sections. Regenerating dMyHC-positive fibers were quantified on entire dMyHC/laminin-γ1/DAPI-stained cross-sections. Only dMyHC-positive fibers that appeared to be healthy were included. Because the antibody used to detect dMyHC was raised in mice, the mean number of dMyHC-positive fibers represents the mean number of dMyHC-positive fibers minus the mean number of fibers that were stained with the secondary antibody alone (that is, 2.8 muscle fibers/cross-section in *dy*^*W*^-L158 mice and 9 fibers/cross-section in *dy*^*W*^/*dy*^*W*^ mice). In all histological quantification experiments, at least four mice from each group were analyzed.

### Western blotting analysis

Triceps brachii and diaphragm muscles were homogenized in radio-immunoprecipitation assay (RIPA) protein extraction buffer (Abcam). A commercial kit (23227; BCA Protein Assay Kit; Pierce Biotechnology Inc., Rockford, IL, USA) was used to determine protein concentrations. Equal amounts of protein (20 μg) were separated in 8% (periostin; 75 to 90 kDa) or 20% (transforming growth factor (TGF)-β; 12 kDa in reducing conditions) SDS–PAGE and transferred to nitrocellulose membrane. The membrane was incubated with antibodies to periostin (RD181045050; BioVendor) or TGF-β (PAB11274; Abnova Corp., Taipei, Taiwan). An antibody to β-actin (#4970; Cell Signaling Technology) was used as loading control. For detection, the appropriate horseradish peroxidase-conjugated antibodies were used, and immunoreactivity was visualized using the enhanced chemiluminescence detection method (32106; Pierce).

### Quantitative real-time PCR

The total RNA of the triceps brachii and diaphragm muscles was extracted (#Z3105; SV Total RNA Isolation System; Promega, Corp., Madison, QI, USA). Samples were calibrated to equal amounts for cDNA synthesis using a commercial kit (#170–8891; iScript cDNA Synthesis Kit; Bio-Rad Laboratories, Inc., Hercules, CA, USA). SYBR Green (4367659; Power SYBR Green PCR Master Mix) was used to perform quantitative PCR in a PCR system (4376600; StepOnePlus™ Real-Time PCR System; both Applied Biosystems, Foster City, CA, USA) with the following primers for TGF-β1 (sense: 5^′^ GACTCTCCACCTGCAAGACCAT 3^′^ and anti-sense: 5^′^ GGGACTGGCGAGCCTTAGTT 3^′^) and on β-actin (sense: 5^′^ CAGCTTCTTTGCAGCTCCTT 3^′^ and anti-sense: 5^′^ GCAGCGATATCGTCATCCA 3^′^) for normalization. The ΔΔ^Ct^ method was used to analyze changes in TGF-β1 mRNA expression relative to WT levels.

### Hydroxyproline assay

Fibrosis in the triceps brachii muscles was measured by assaying for the exclusive collagen-specific modified amino acid hydroxyproline. Tendons were carefully removed before muscles were speed-dried under vacuum and sent for amino acid analysis (Analytical Research Services; Bern, Switzerland) as described previously [19]. There, muscles were hydrolyzed, then evaporated to dryness, and resuspended in 0.1% trifluoroacetic acid. Amino acids were determined by a routine method [43] using high-performance liquid chromatography (HPLC) to identify and quantify the amino acid hydroxyproline. The relative hydroxyproline amount was assessed with reference to the total amount of amino acids.

### Notexin-induced muscle damage

The tibialis anterior (TA) muscle of 5 week-old mice was injured by injection of 15 μl notexin (50 μg/ml; Sigma-Aldrich) as described previously [21]. Mice were killed 5 days after injection, and muscles were isolated and processed as described above.

### Body weight, locomotion, and grip strength

Body weight was measured at the age of 7 weeks before mice were sacrificed. Locomotive behavior was recorded by placing the mice into a new cage and measuring motor activity (walking, digging, stand-ups on hindlegs) for 10 minutes [25]. Grip strength was evaluated by placing the animals onto a vertical grid, and measuring the time until they fell down; the cut-off time was 180 seconds. In all tests, at least 12 animals of each genotype were analyzed, and values were normalized to those obtained from WT animals.

### Statistical analysis

To compare the different genotypes, *P*-values were calculated using the one-way ANOVA test.

## Results

### Angiotensin II receptor type 1 (AT1) signaling and TGF-β levels are increased in *dy*^*W*^*/dy*^*W*^ mice

**Figure 1:**
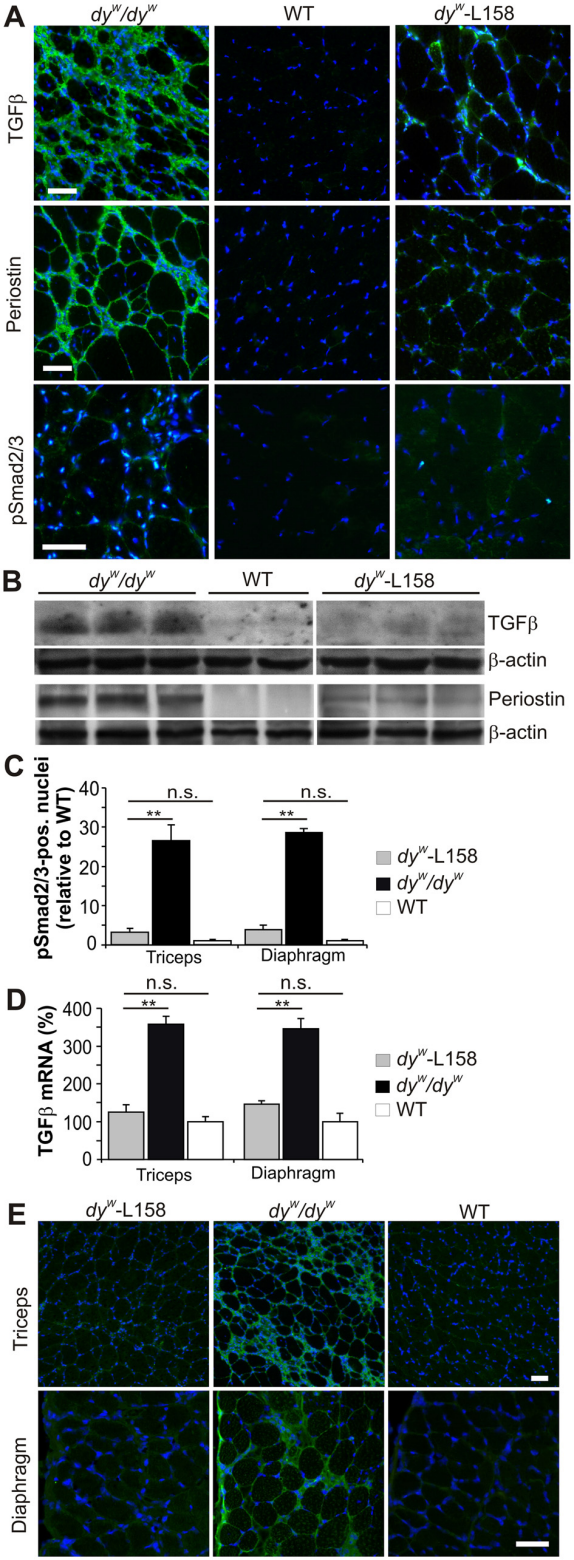
**L-158809 lowered angiotensin II receptor type 1 (AT1) -mediated transforming growth factor (TGF)-β levels in the muscles of *dy***^***W***^***/dy***^***W ***^**mice. (A) **AT1-mediated TGF-β levels were increased in muscles of *dy*^*W*^*/dy*^*W *^mice and decreased after L-158809 administration. Triceps cross-sections of *dy*^*W*^*/dy*^*W *^mice showed expression of TGF-β (green) and periostin (green), and nuclear accumulation of pSmad2/3 (green), which is a downstream target of AT1. Oral L-158809 treatment of *dy*^*W*^*/dy*^*W *^mice (*dy*^*W*^-L158) reduced expression of all three proteins. Nuclei were visualized with 4",6"-diamidino-2-phenylindole hydrochloride (DAPI) (blue). **(B)** Western blotting analysis of triceps muscles confirmed increased protein expression levels of periostin (75 to 85 kDa) and TGF-β (12 kDa, reduced) in *dy*^*W*^*/dy*^*W *^mice as well as the reduction after L-158809 treatment. β-actin was used as a loading control. **(C) **The number of pSmad2/3-positive nuclei was more than 25-fold increased in cross-sections of *dy*^*W*^*/dy*^*W *^muscle compared with wild-type (WT) muscles, and was significantly reduced by L-158809 to levels 2-fold (triceps) and 2.6-fold (diaphragm) those of WT mice, which was not significant (n ≥ 4). **(D)** Quantitative real-time PCR showed an increase of approximately 3.5-fold in TGF-β1 mRNA levels in both *dy*^*W*^*/dy*^*W *^triceps and diaphragm muscles, which was reduced to nearly WT levels by L-158809 (n ≥ 4). **(E) **Thrombospondin-1 (green) was increased in both triceps and diaphragm muscles of *dy*^*W*^*/dy*^*W *^mice, and was minimized by L-158809 administration. All values represent the mean ± SEM. One-way ANOVA: **P ≤ 0.001; * *P* ≤ 0.05; n.s. (non-significant) *P *> 0.05. Scale bar = 50 μm.

In the first set of experiments, we tested whether TGF-β levels were increased in muscles of laminin-α2-deficient *dy*^*W*^*/dy*^*W*^ mice. We found that TGF-β (Figure 1, green staining) was present in the perimysium and endomysium of triceps muscle from *dy*^*W*^*/dy*^*W*^ mice, but was absent in WT mice (Figure 1A). Accordingly, expression of periostin and nuclear accumulation of phosphorylated Smad2/3 complexes (pSmad2/3), both downstream mediators of TGF-β signaling, were seen in *dy*^*W*^*/dy*^*W*^ but not in WT triceps (Figure 1A). Western blotting analysis confirmed the presence of TGF-β and periostin in *dy*^*W*^*/dy*^*W*^ muscles (Figure 1B, first panel).

To test whether L-158809 decreased TGF-β levels, we treated *dy*^*W*^*/dy*^*W*^ mice with L-158809 for 3 weeks. Stains and immunoblots of triceps muscles from the treated *dy*^*W*^*/dy*^*W*^ mice (*dy*^*W*^-L158) showed a strong reduction in TGF-β and periostin (right panels, Figure 1A and Figure 1B). Correspondingly, nuclear accumulation of pSmad2/3 complexes was 25 times higher in *dy*^*W*^*/dy*^*W*^ than in WT muscles, but was reduced to almost WT levels after L-158809 treatment (Figure 1A, right panel; Figure 1C). The suppression of TGF-β by L-158809 was also seen for mRNA levels, which were less than 50% of those in *dy*^*W*^*/dy*^*W*^ mice (Figure 1D). Importantly, TSP-1, which is increased by AT1 signaling [44], and has been shown to mediate activation of TGF-β in cardiac muscle and kidney [45], was present in the extracellular matrix of *dy*^*W*^*/dy*^*W*^ mice, but was strongly reduced in L-158809-treated *dy*^*W*^*/dy*^*W*^ triceps and diaphragm muscle (Figure 1E). These results indicate that AT1-mediated TGF-β signaling is increased in MDC1A , and that L-158809 dampens this pathway.

### Angiotensin II type 1 receptor antagonism improves fibrosis, inflammation, and overall histology

**Figure 2:**
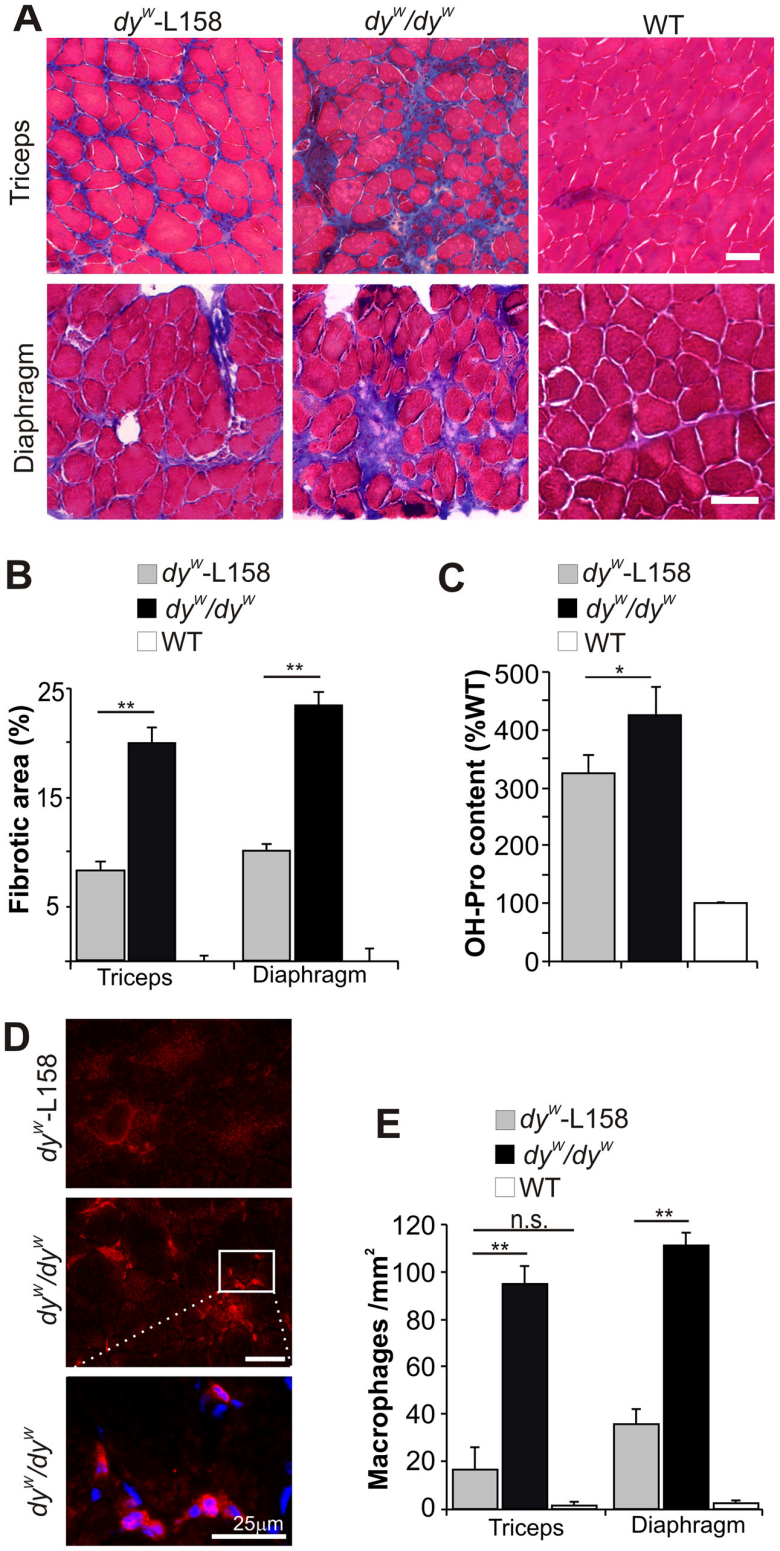
**L-158809 reduces fibrosis and inflammation in the muscles of *dy***^***W***^***/dy***^***W ***^**mice. (A) **Masson trichrome staining showed higher collagen content (blue) in triceps and diaphragm muscles of *dy*^*W*^*/dy*^*W *^when compared with *dy*^*W*^-L158 mice. **(B) **Average amount of fibrotic area relative to wild-type (WT) muscles. L-158809 treatment more than halved the fibrotic area in triceps and diaphragm muscles (n ≥ 4). **(C) **Measurement of the amount of hydroxyproline as an indicator of fibrosis. L-158809 treatment of *dy*^*W*^*/dy*^*W *^mice reduced the hydroxyproline amount in triceps muscle (n = 3). **(D)** F4/80 staining of macrophages in diaphragm muscles. **(E) **L-158809 treatment reduced the number of macrophages in muscles of *dy*^*W*^*/dy*^*W *^mice (n ≥ 4). All values represent the mean ± SEM. One-way ANOVA: **P ≤ 0.001; * *P *≤ 0.05; n.s. (non-significant) *P *> 0.05. Scale bar = 50 μm.

To test for a beneficial effect of L-158809, we treated *dy*^*W*^*/dy*^*W*^ mice starting at the age of 3 weeks and continuing until the age of 7 weeks. First, we analyzed the potential of L-158809 to reduce fibrosis and inflammation in triceps and diaphragm muscle of *dy*^*W*^*/dy*^*W*^ mice. Masson trichrome staining showed a more prominent replacement of muscle tissue with non-muscle cells in untreated *dy*^*W*^*/dy*^*W*^ mice than in mice treated with L-158809 (Figure 2A). The blue color, indicative of collagen, suggests that the non-muscle cells found in untreated muscles represent mainly fibrotic tissue (Figure 2A). The fibrotic area measured in cross-sections of triceps and diaphragm muscle of *dy*^*W*^*/dy*^*W*^ mice treated with L-158809 was less than 50% of that seen in untreated mice (Figure 2B). As an independent measure of fibrosis, we also determined the hydroxyproline content in triceps muscles. In agreement with the histological measurements, L-158809 reduced the hydroxyproline content in *dy*^*W*^*/dy*^*W*^ muscles (Figure 2C). Because TGF-β plays an important role in regulating skeletal-muscle inflammation [38,46], we also assessed inflammation using the F4/80 antibody that recognizes activated macrophages (Figure 2D,E). Macrophages were mainly located in areas where muscle fiber degeneration is taking place (Figure 2D). In untreated *dy*^*W*^*/dy*^*W*^ muscles, around 100 macrophages were found per mm^2^, whereas L-158809 reduced this number by six times in triceps and by three times in diaphragm muscle (Figure 2E). These data provide evidence that L-158809 inhibits and thus minimizes TGF-β-induced fibrosis and inflammation in muscles of *dy*^*W*^*/dy*^*W*^ mice.

**Figure 3:**
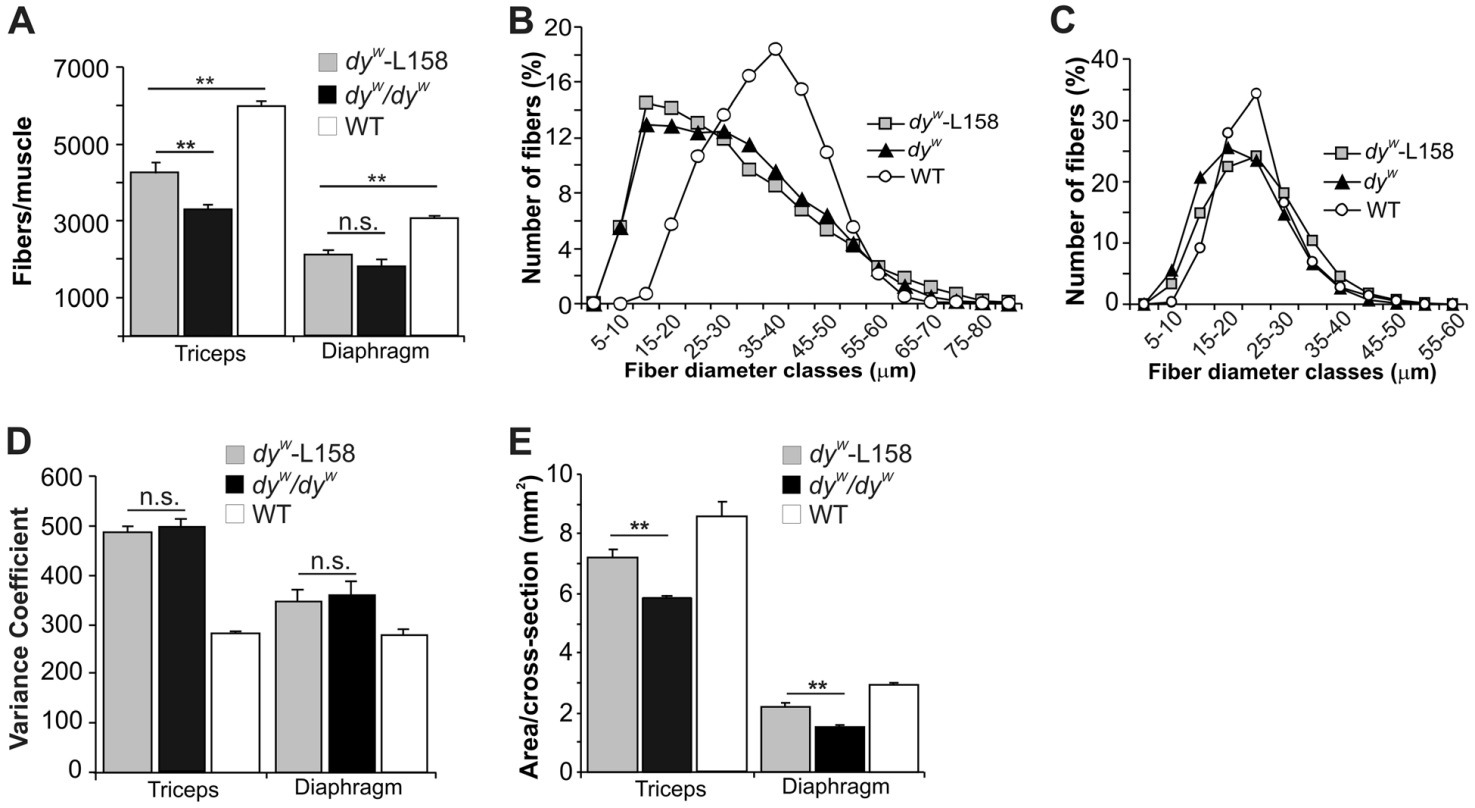
**L-158809 reduced muscle-fiber loss but did not change fiber-size distribution in *dy***^***W***^***/dy***^***W ***^**muscles. (A) **L-158809 (*dy*^*W*^-L158) stabilized the loss of muscle fibers in *dy*^*W*^*/dy*^*W *^triceps muscle, but only tended to reduce fiber loss in the diaphragm (n = 6). **(B,C) **L-158809 did not normalize the shift of fiber sizes to smaller fibers, either **(B) **in triceps or **(C) **in diaphragm muscle of *dy*^*W*^*/dy*^*W *^mice (n ≥ 4). **(D) **The minimal Feret`s diameter coefficient was unchanged by L-158809 (n ≥ 4). **(E) **L-158809 increased the cross-sectional area in both mid-belly triceps (P = 0.0007) and diaphragm (P = 0.0024) muscles (n ≥ 4). All values represent the mean ± SEM. P-values (one-way ANOVA): **P ≤ 0.001; * *P *≤ 0.05; n.s. (non-significant) *P *> 0.05.

We next investigated whether the L-158809-mediated reduction of fibrosis also ameliorated other histological hallmarks of MDC1A. A prominent feature of laminin-α2-deficient muscles is the loss of muscle fibers, which is probably due to muscle degeneration and the difficulty in muscle regeneration [18]. Indeed, the number of muscle fibers in *dy*^*W*^*/dy*^*W*^ mice was much lower than in WT mice, with the triceps and diaphragm, respectively losing 46% and 40% of their fibers (Figure 3A). L-158809 decreased this fiber loss in triceps muscle, and increased the fiber number to 68% of WT. In diaphragm, L-158809 showed a similar trend, but this was not significant (Figure 3A). Further, L-158809 did not affect fiber size distribution in *dy*^*W*^*/dy*^*W*^ triceps muscle (Figure 3B). In diaphragm, where muscle fiber-size distribution was only slightly shifted to smaller diameters in *dy*^*W*^*/dy*^*W*^ mice, L-158809 shifted fiber-size distribution only slightly towards the values in WT (Figure 3C). Accordingly, the minimal Feret’s variance coefficient [42] was increased in *dy*^*W*^*/dy*^*W*^ muscles but was not corrected by L-158809 (Figure 3D). However, because of the higher number of muscle fibers in L-158809-treated *dy*^*W*^*/dy*^*W*^ mice, the mean cross-section area of the muscle was significantly increased (Figure 3E). Thus, L-158809 partially prevents the loss of muscle fibers, but does not significantly affect the diameter of *dy*^*W*^*/dy*^*W*^ muscle fibers.

### L-158809 strongly improves spontaneous and injury-induced muscle regeneration

**Figure 4:**
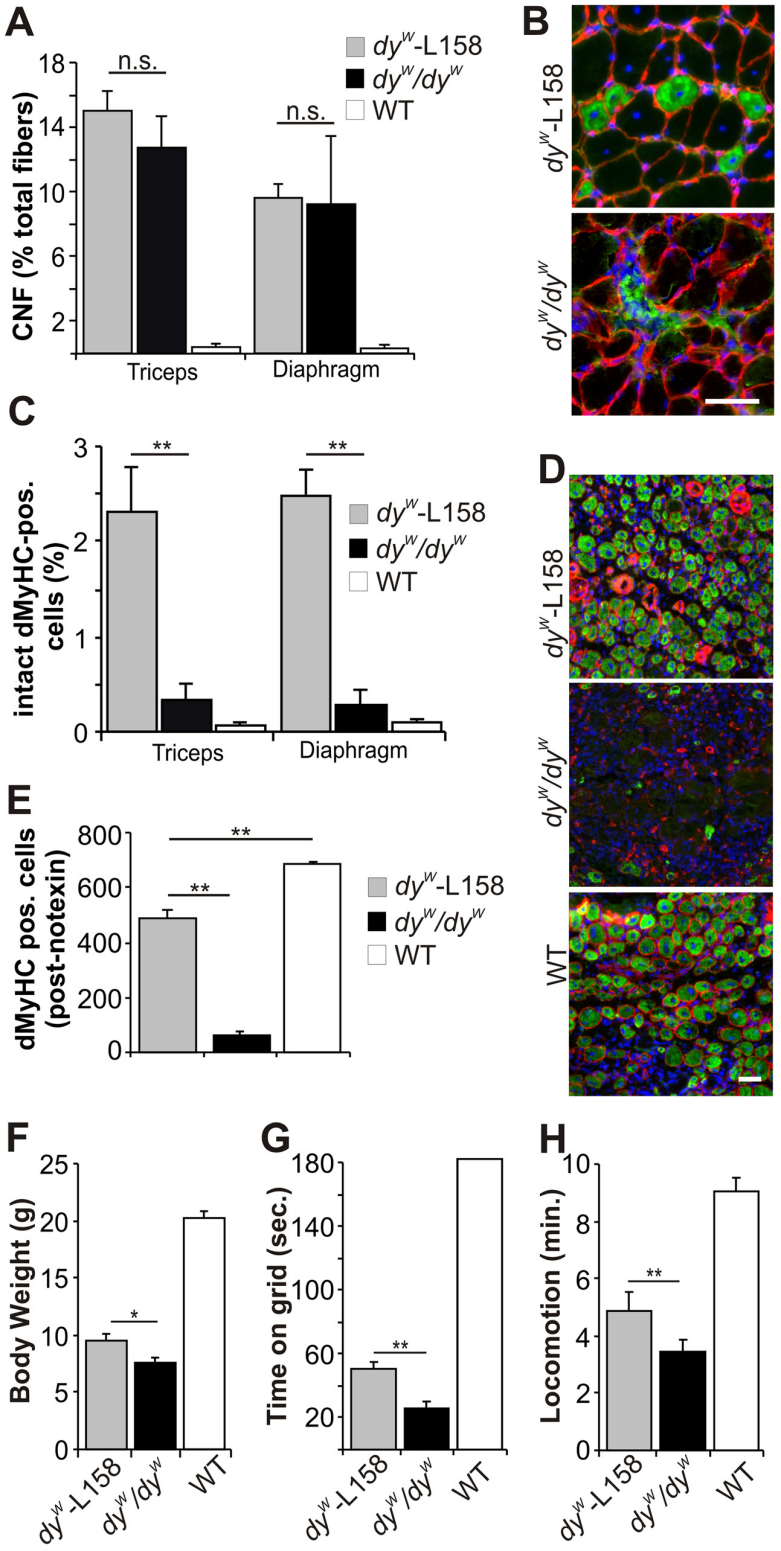
**L-158809 improved muscle regeneration, body weight, locomotion, and muscle strength in *dy***^***W***^***/dy***^***W ***^**mice. (A) **L-158809 (*dy*^*W*^-L158) did not significantly affect the number of centrally nucleated fibers (CNF) in either triceps or diaphragm muscle of *dy*^*W*^*/dy*^*W *^mice (n ≥ 4). **(B) **Staining of diaphragm muscle for the regeneration marker developmental myosin heavy chain (dMyHC; green), the basement membrane marker laminin-γ1 (red), and the nuclear marker 4",6"-diamidino-2-phenylindole hydrochloride (DAPI) (blue). L-158809 enabled damaged *dy*^*W*^*/dy*^*W *^muscle fibers to regenerate, whereas in non-treated *dy*^*W*^*/dy*^*W *^muscle many dMyHC-expressing cells continued degenerating. **(C)** L-158809 application strongly increased the number of intact regenerating muscle fibers in *dy*^*W*^*/dy*^*W *^mice (n ≥ 4). **(D) **Tibialis anterior (TA) muscle stained for dMyHC (green), laminin-γ1 (red), and DAPI four days after notexin-induced injury. L-158809 increased the regenerative capacity of damaged *dy*^*W*^*/dy*^*W *^muscle. **(E) **The number of dMyHC-expressing fibers in TA muscle of *dy*^*W*^/*dy*^*W *^mice 5 days after notexin injection was more than 8 times higher in L-158809 treated *dy*^*W*^*/dy*^*W *^mice but was still lower than in controls (n = 3). **(F) **L-158809 increased the average body weight of *dy*^*W*^*/dy*^*W *^mice from 7.6 g to 9.5 g, although age-matched wild-type (WT) mice weigh more than 20 g. (n ≥ 12). **(G)**. L-158809 doubled the time a *dy*^*W*^*/dy*^*W *^mouse could hold itself on a vertical grid, from 26 seconds to 51 seconds (P = 0.0001) (n ≥ 14). **(H) **L-158809 increased the time *dy*^*W*^*/dy*^*W *^mice explored an unknown surrounding (P = 0.0004) within 10 minutes (n ≥ 12). All values represent the mean ± SEM (n ≥ 4). One-way ANOVA: **P ≤ 0.001; * *P *≤ 0.05; n.s. (non-significant) *P *> 0.05. Scale bar = 50 μm, if not indicated differently.

The muscles of d*y*^*W*^*/dy*^*W*^ mice are strongly impaired in muscle regeneration [18,19,21]. In addition, increased TGF-β activity leads to failure of muscle regeneration [1]. Therefore, L-158809 may improve regeneration of *dy*^*W*^*/dy*^*W*^ muscle. However, the number of centrally nucleated fibers, which are indicative of ongoing degeneration/regeneration, were not significantly increased after L-158809 administration (Figure 4A). By contrast, staining of diaphragm muscle with the regeneration marker dMyHC (Figure 4B, green) indicated that more fibers regenerated after L-158809 treatment of *dy*^*W*^*/dy*^*W*^ mice. Muscle fibers expressing dMyHC in the d*y*^*W*^-L158 mice appeared to have a proper basement membrane as indicated by laminin-γ1 staining (red), whereas in non-treated *dy*^*W*^*/dy*^*W*^ mice, dMyHC staining was often punctated and the basement membrane appeared disrupted, indicating that the fibers fail to complete regeneration (Figure 4B). In both triceps and diaphragm muscle, L-158809 clearly increased the number of intact dMyHC-positive fibers (Figure 4C), suggesting that regeneration was improved by L-158809.

To test the effect of L-158809 on the regenerative capacity, the TA muscle was injured by injection of notexin. Four days after injury, only a few dMyHC-positive fibers were found in *dy*^*W*^*/dy*^*W*^ mice, whereas many were present after L-158809 treatment or in WT muscle (Figure 4D). The number of regenerating fibers increased from 61 ± 14 in untreated to 490 ± 25 in L-158809-treated *dy*^*W*^*/dy*^*W*^ TA muscle (Figure 4E). Thus, L-158809-mediated TGF-β inhibition improved the regeneration capacity in muscles of *dy*^*W*^*/dy*^*W*^ mice. Because a similar improvement in muscle regeneration in *dy*^*W*^/*dy*^*W*^ mice has also been reported for treatments that inhibited apoptosis [47], we also tested whether L-158809 would influence the number of apoptotic nuclei. However, we could not detect any effect of this treatment on the number of terminal deoxynucleotidyl transferase-mediated dUTP nick end labeling-positive myonuclei (see Additional file 1: Figure S1), indicating that L-158809 does not affect this pathway.

### Effect of L-158809 on overall health of *dy*^*W*^*/dy*^*W*^ mice

Finally, we tested whether there was any improvement in motor function and overall health in L-158809-treated *dy*^*W*^*/dy*^*W*^ mice, and found that L-158809 increased the body weight of *dy*^*W*^*/dy*^*W*^ mice, by 2 g (Figure 4F). However, age-matched WT animals still weighed more than twice as much. The muscle strength of *dy*^*W*^*/dy*^*W*^ mice, measured as the time the animals could stay on a vertical grid, was doubled by treatment with L-158809 from 26 seconds to 51 seconds (Figure 4G); however, the WT animals far exceeded the time measured with L-158809-treated *dy*^*W*^*/dy*^*W*^ mice, normally staying more than 180 seconds on the grid. In the 10-minute open-field locomotion test L-158809-treated mice were more active and explored the new surroundings for 5 minutes, whereas untreated MDC1A mice were active only for 3.5 minutes (Figure 4H). Thus, the ameliorating effect of L-158809 on muscle histology is sufficient to improve body and muscle condition of *dy*^*W*^*/dy*^*W*^ mice.

## Discussion

Muscular dystrophies are caused by mutations in many different genes but they are often accompanied by a strong inflammatory and fibrotic response, which might be triggered by similar signaling pathways. For example, TGF-β signaling has been shown to be upregulated in several muscle diseases [8-10], and is known to inhibit myoblast differentiation and promote fibrosis, and also to impair regeneration by inhibiting satellite cell activation, proliferation, and differentiation [3,4].

In this study, we found that TGF-β signaling, including increased levels of TGF-β and of the phosphorylated forms of Smad2 and Smad3, was also enhanced in the severe *dy*^*W*^*/dy*^*W*^ mouse model of MDC1A (Figure 1). These data are in good agreement with previous reports that TGF-β levels are also upregulated in the muscles of human patients with MDC1A [38] and in the mild *dy*^*2J*^/*dy*^*2J*^ mouse model [37]. As in the study on *dy*^*2J*^/*dy*^*2J*^ mice, we found an increase in the TGF-β-activating protease TSP-1, indicating that the elevation of TGF-β is probably caused by the activation of AT1, as production of TSP-1 precedes TGF-β activation in this pathway [1,2,44,45]. Indeed, inhibition of AT1 signaling by oral administration of L-158809 for only 3 weeks was sufficient to lower the amount of TSP-1 in muscle basement membrane and to normalize TGF-β levels and Smad2/3 phosphorylation (Figure 1). These experiments provide strong evidence that the AT1-TSP-TGF-β axis is also activated in MDC1A and they are in accordance with the decreased TGF-β levels and Smad2/3 phosphorylation seen in *dy*^*2J*^/*dy*^*2J*^ mice [37]. Previous work in kidney [44] has shown that the AT1-mediated increase in TSP-1 involves the MAPK pathway. This pathway may also be active in MDC1A, as the MAPKs ERK1/2, p38 and JNK are all increased in *dy*^*2J*^*/dy*^*2J*^ mice, and this increase can be inhibited by losartan [37].

### Angiotensin II type 1 receptor antagonists have a strong effect on muscle regeneration in *dy*^*W*^*/dy*^*W*^ mice

Increased TGF-β signaling inhibits activation of satellite cells [48] and promotes differentiation of myogenic into fibrotic cells in injured skeletal muscles [3], indicating that TGF-β-dependent fibrosis is associated with impaired muscle regeneration. In mouse models of MFS and Duchenne muscular dystrophy (DMD), losartan treatment restored the muscle-regeneration capacity after cardiotoxin-induced muscle injury [1]. In mice with sarcopenia, losartan restored the necessary downregulation of Pax7 and MyoD and upregulation of p21 and myogenin, which are required for successful completion of regeneration of injured muscle, by modulating TGF-β signaling both via Smad2/Smad3 phosphorylation and via activation of MAPKs [6,13]. Muscle regeneration is largely impaired in MDC1A, and we have recently shown that the main effect of anti-apoptosis treatment in *dy*^*W*^*/dy*^*W*^ mice is the prevention of cell death during the regeneration process [47]. In addition, this previous work also provided evidence that the cell death prevention by anti-apoptosis treatment had a synergistic effect when combined with the re-establishment of the connection between the basement membrane and the muscle membrane by mini-agrin [47].

In the current study, we found evidence that AT1 blockade by L-158809 also ameliorated the muscle regeneration capacity in *dy*^*W*^*/dy*^*W*^ mice. Importantly, we found that L-158809 treatment enabled newly formed muscle fibers to stay intact and to complete regeneration, whereas non-treated *dy*^*W*^*/dy*^*W*^ fibers started to regenerate, but then turned into fibrotic cells. The beneficial effect of L-158809 on regeneration is superior to that seen in *dy*^*W*^*/dy*^*W*^ mice treated with an apoptosis inhibitor alone [47], and reached almost the same efficacy as the combination of apoptosis inhibition and mini-agrin [47]. Our observation of a relatively strong beneficial effect of AT1 antagonists on muscle regeneration is in good accordance with the finding in mice with sarcopenia, in which losartan enabled the regeneration of skeletal muscle by fostering satellite cells to transit from a proliferation stage into the differentiation process [13]. Our finding that L-158809 treatment lowered the number of centrally located myonuclei suggests that AT1 blockade might also prevent necrosis of muscle fibers.

### Effect of Angiotensin II receptor type 1 antagonist on fibrosis, inflammation, and overall muscle function

Previous work has provided evidence that AT1 antagonists can ameliorate disease in mouse models of MFS and DMD and in mice with sarcopenia [1,13-15]. We found that 4 weeks of L-158809 treatment decreased fibrosis from 19.7% to 8.1% in triceps and from 23.3% to 10% in diaphragm muscle, and thereby prevented the characteristic inflammatory response. Similarly, 12 weeks of losartan treatment reduced the fibrotic area in *dy*^*2J*^/*dy*^*2J*^ by approximately 4% [37]. In addition, L-158809 treatment in *dy*^*W*^*/dy*^*W*^ mice corrected some of the histological hallmarks of the condition, such as fiber loss and muscle area. In contrast to its effects in *mdx* mice, L-158809 did not correct the shift of the fiber-size distribution to smaller fibers in *dy*^*W*^*/dy*^*W*^ mice. This phenomenon was due to the presence of many newly formed regenerating (dMyHC-positive) fibers that were not grown to full size. Treatment with L-158809 also resulted in a significant functional improvement in skeletal muscle, manifesting as increased locomotor activity and increased grip strength. Interestingly, an improvement of the hind-limb and fore-limb strength was also seen in the *dy*^*2J*^*/dy*^*2J*^ mouse model of MDC1A [37].

### Therapeutic potential of the different treatment options

The highest potential to restore function in mouse models for MDC1A is the body-wide, transgenic expression of laminin-α1, which largely cures the disease [26,27]. However, because the cDNA encoding laminin-α1 is more than 9 kb in size, its use in gene therapy is challenging, and no successful attempts in creating smaller versions of the functional protein have been reported. Another very effective treatment option is the muscle-specific overexpression of a miniaturized form of agrin (mini-agrin), specifically designed to structurally reconnect the cell surface protein α-dystroglycan to laminins in the basement membrane [19,21,25]. Finally, overexpression of insulin-like growth factor (IGF)-1 also ameliorates disease and prolongs life span [49], but its efficacy is clearly lower than that of mini-agrin. This lower efficacy is probably based on the fact that IGF-1 interferes with rather late events in the course of the disease, whereas mini-agrin tackles the structural deficits that are the primary cause of the muscular dystrophy. Recombinant AAV-based gene therapy with mini-agrin was shown to be successful in *dy*^*W*^*/dy*^*W*^ mice [28]. However, the use of gene therapy in clinics has been rather slow, and thus delivery of mini-agrin to the muscles of patients with MDC1A remains difficult.

This difficulty in translating gene therapy into clinics has led to attempts to interfere with disease progression using protein therapy and pharmacological compounds. An interesting approach that has been shown to be efficacious in *dy*^*W*^*/dy*^*W*^ mice is the systemic delivery of laminin-111 (heterotrimer of the α1, β1, and γ1 chains). In those studies, laminin-111 reached the skeletal muscles, improved muscle histology, and prolonged life span [50]. Pharmacological approaches include the use of the apoptosis inhibitors omigapil [30] or of doxycycline or minocycline [31] to treat *dy*^*W*^/*dy*^*W*^ mice. In both studies, there was a clear effect on functional parameters and on the longevity of the mice; however, the efficacy of either treatment was much lower than in *dy*^*W*^*/dy*^*W*^ mice expressing mini-agrin [47].

Another consequence of muscular dystrophies is a severe muscle-wasting. Preservation of muscle mass depends on the proper balance between protein synthesis and protein degradation [51]. Thus, muscle wasting can be due to a prevalence of protein degradation, and based on this idea, pharmacological inhibition of the two main systems involved in protein degradation, the proteasomal and the autophagic pathway, has been tested in the preclinical mouse models for MDC1A. Again, interference with either of those pathways ameliorated disease progression [33,34]. It is, however, questionable if such an approach is a good option for the treatment of patients with MDC1A, as inhibition of proteosomal and autophagosomal degradation has a strong likelihood of causing severe side-effects.

In the current study, we provide evidence that AT1 antagonists also provide benefit to *dy*^*W*^*/dy*^*W*^ mice. Our results are similar to those of other researchers who tested inhibition of TGF-β-induced fibrosis by intraperitoneal injection of halofuginone in the milder *dy*^*2J*^*/dy*^*2J*^ mouse model of MDC1A [36]. Halofuginone blocked TGF-β-mediated collagen synthesis, prevented muscle fibrosis, and improved the performance of both muscles and animals. In addition, the same group also used losartan to block TGF-β-signaling and this also showed benefit in *dy*^*2J*^*/dy*^*2J*^ mice [37]. Thus, our work is an important confirmation of efficacy of AT1 antagonists in the more severe mouse model of MDC1A.

It is difficult to compare studies that were performed in *dy*^*W*^*/dy*^*W*^ mice with those using the more severe *dy*^*3K*^*/dy*^*3K*^ mouse model or the much milder *dy*^*2J*^*/dy*^*2J*^ model. As our current study used *dy*^*W*^*/dy*^*W*^ mice, it can be compared with those using the pharmacological apoptosis inhibitors omigapil or doxycycline. A detailed analysis of those studies indicates that all of the treatments are of comparable benefit to the mice. For example, they all increased body weight by approximately 2 g and in the open-field locomotion test, both L-158809 and omigapil increased activity time by 1.5 minutes. Treatment with omigapil and doxycycline approximately doubled the lifespan of *dy*^*W*^*/dy*^*W*^ mice. Because of changes in the laws regarding animal testing, we could not study the survival in a large cohort of animals and could only record age at death in four cases of L-158809-treated *dy*^*W*^*/dy*^*W*^ mice. Nevertheless, all four mice lived at least 5 weeks longer than the untreated *dy*^*W*^*/dy*^*W*^ mice (~10 weeks in average). Thus, as L-158809 or losartan does not act on apoptosis (see Additional file 1: Figure S1), it remains to be tested whether a combination of losartan and omigapil could provide an additive benefit in *dy*^*W*^*/dy*^*W*^ mice. The fact that losartan is well tolerated in all age groups and that omigapil has proven to be safe in clinical trials of patients with Parkinson’s disease and those with amyotrophic lateral sclerosis [52,53], makes such a combination attractive for further clinical trials.

## Conclusion

In this study, we investigated the efficacy of AT1 antagonists in the most widely used mouse model of MDC1A. The benefits of the therapy included decreased fibrosis and inflammation, improved muscle regeneration (which leads to preservation of muscle-fiber number), and improved locomotion and grip strength. The AT1 antagonist losartan is an approved anti-hypertensive drug that is well tolerated, including in children. And, importantly, losartan harbors fewer potential side-effects than any other pharmacological options that have been tested to date in preclinical mouse models of MDC1A. Therefore, AT1 blockade could provide a supportive treatment that alleviates some of the pathology in patients with MDC1A in the near future.

## Abbreviations

AAV: Adeno-associated virus; AT1: Angiotensin II receptor type 1; DMD: Duchenne muscular dystrophy; dMyHC: Developmental myosin heavy chain; *dy*^*W*^/*dy*^*W*^: Laminin-α2-deficient MDC1A mouse model; *dy*^*W*^-L158: L-158809-treated *dy*^*W*^*/dy*^*W*^ mice; *Fbn1*C1039G/+: MFS mouse model; *mdx*: DMD mouse model; MDC1A: Laminin-α2-deficient congenital muscular dystrophy; MFS: Marfan syndrome; Mini-agrin: Miniaturized form of agrin specifically designed to contain laminin and α-dystroglycan binding domains; WT: Wild-type.

## Competing interests

The authors declare no competing interests

## Authors' contributions

SM performed and analyzed the experiments. SL assisted in some of the experiments, and provided scientific input. SM and MAR conceived and designed the study, and wrote the manuscript. All authors read and approved the final manuscript.

## Supplementary Material

Additional file 1 Figure S1. Click here for file
