# Supplementary material for: Angiotensin II type 1 receptor antagonists alleviate muscle pathology in the mouse model for laminin-α2-deficient congenital muscular dystrophy (MDC1A)
Source: Skelet Muscle. 2012 Sep 3;2:18. doi: 10.1186/2044-5040-2-18 (PMC3598380; doi:10.1186/2044-5040-2-18)
Supplement: Additional file 1 — Figure S1. [file 2044-5040-2-18-S1.pdf]

Figure S1

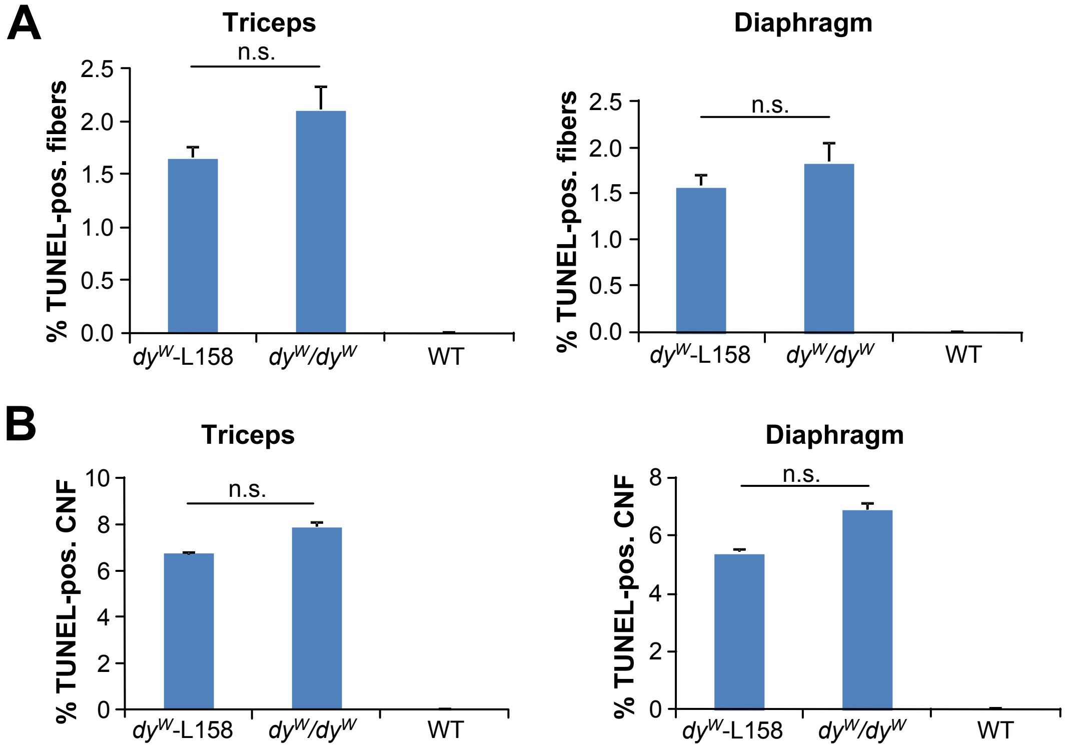

Figure S1. Quantification of TUNEL staining that recognizes apoptotic nuclei in triceps and diaphragm cross-sections of *dy*^{W}-L158, *dy*^{W}/*dy*^{W} and WT mice. (**A**) Percentage of apoptotic fibers. There is a non-significant trend to less fibers containing TUNEL-positive nuclei in *dy*^{W}-L158 (triceps = 1.65%, diaphragm = 1.6%) when compared to *dy*^{W}/*dy*^{W} (triceps = 2.1%, diaphragm = 1.85%) muscles. (**B**) Percentage of regenerating fibers with centralized nuclei undergoing apoptosis. L158809 treatment tends to reduce the number of TUNEL-positive centralized nuclei in *dy*^{W}/*dy*^{W} mice (*dy*^{W}/*dy*^{W}: triceps = 7.8%, diaphragm = 6.8%, *dy*^{W}-L158: triceps = 6.7%, diaphragm = 5.4%). All values represent the mean ±SEM. N . N≥4. P-values (one-way ANOVA): n.s. p>0.05.
